# PPARγ/Pgc-1α-Fndc5 pathway up-regulation in gastrocnemius and heart muscle of exercised, branched chain amino acid diet fed mice

**DOI:** 10.1186/s12986-018-0298-3

**Published:** 2018-08-29

**Authors:** Navid Abedpoor, Farzaneh Taghian, Kamran Ghaedi, Iman Niktab, Zahra Safaeinejad, Farzaneh Rabiee, Sommayeh Tanhaei, Mohammad Hossein Nasr-Esfahani

**Affiliations:** 1 Department of Physical Education and Sports Science, Isfahan (Khorasgan) Branch, Islamic Azad University, Isfahan, Iran; 2 Department of Biology, Faculty of Sciences, University of Isfahan, Isfahan, Iran; 3 Department of Cellular Biotechnology, Cell Science Research Center, Royan Institute for Biotechnology, ACECR, Royan Street, Salman Street, Isfahan, 816513-1378 Iran

**Keywords:** Aerobic exercise, Branched-chain amino acids, Catabolism, *Fndc5*, Irisin, *Pgc-1α*, *PPARγ*

## Abstract

**Background:**

Previous studies have revealed the inductive effect of branched-chain amino acids (BCAAs) catabolism on fatty acid oxidation and metabolism, especially in muscle cells. In the present investigation, we have attempted to address whether a combination of BCAAs supplement consumption with aerobic exercise could elaborate the expression of *PPAR*γ, *Pgc-1α* and *Fndc5* genes in gastrocnemius muscle and heart tissue of male C57BL/6 mice.

**Methods:**

Thirty-six young male mice with an average weight of 18 ± 2 g were selected. Mice were randomly assigned to 6 groups: 20 mg/mL of BCAAs consumption with simultaneous exercise-training, 60 mg/mL of BCAAs consumption with simultaneous exercise-training, exercise-trained with no BCAAs consumption group, 20 mg/mL BCAAs without exercise-training, 60 mg/mL BCAAs without exercise-training, and untrained mice without BCAAs consumption.

**Results:**

The findings showed a combination of 20 mg/mL BCAAs with aerobic exercise significantly increased *Fndc5*, *PPARγ*, *Pgc-1α* gene expression in skeletal muscles although, circulating Irisin levels remained unchanged (*p* < 0.05). Interestingly, plasma urea and lactate levels were significantly increased in 60 mg/mL BCAAs administrated mice which performed exercised (*p* < 0.05). Two-way analysis of variance (ANOVA) was used to examine significant difference between groups and sedentary group.

**Conclusions:**

Results showed inductive effect of 20 mg/mL BCAAs on expression levels of *Fndc5*, *PPARγ*, *Pgc-1α in* gastrocnemius muscle similar with counterparts in heart tissue. Of note, higher serum irisin levels were detected after 20 mg/mL BCAAs supplementation coincided with the exercise.

**Graphical abstract:**

An Overview on supplemantaion of branched chain amoinoacids on metablism of skeletal muscle and heart
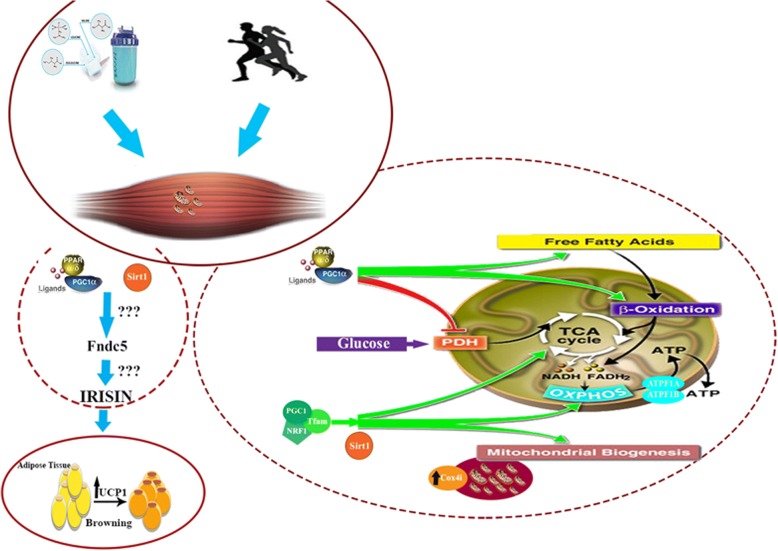

**Electronic supplementary material:**

The online version of this article (10.1186/s12986-018-0298-3) contains supplementary material, which is available to authorized users.

## Background

Branched Chain Amino Acids (BCAAs), including Leucine, Isoleucine, and Valine, are essential amino acids that body is unable to synthesize and needs to be provided by the diet. BCAAs play a significant role in energy homeostasis as they are important for the maintenance of skeletal muscle [1]. Restorative effects of BCAAs on muscle soreness and fatigue are already well recognized [2, 3]. A great proportion of BCAAs metabolism occurs in skeletal muscle, where metabolism is regulated. In muscle, BCAAs stimulate mammalian target of rapamycin complex 1 (mTORC1) and protein synthesis and inhibit protein catabolism [4–6]. Moreover, BCAAs improve aerobic capacity and threshold of anaerobic activities in human Previous studies have shown that consumption of BCAAs increases lipid metabolism and reduces concentration of skeletal muscle triglycerides Interestingly, dietary BCAAs supplementation has intensified lifetime via promotion of mitochondrial biogenesis and enhancement in *Sirtuin 1* (*Sirt1*) expression besides a reduction in oxidative damage [7–11]. Therefore BCAAs can be beneficial for energy expenditure and health status.

Recent studies revealed substantial role of fibronectin type III domain containing 5 (FNDC5) and its secretory type, irisin, in energy metabolism and regulation [12, 13]. *FNDC5* expression is induced by exercise [12, 13]. Huh et al. have shown that plasma irisin and expression of *FNDC5* are increased in response to acute exercise [15]. Of significant worth is the positive association between oxidative components and ROS with irisin content in trained mice [16].

Another key player in regulation of metabolism is peroxisome proliferator-activated receptor gamma (*PPAR*γ)/peroxisome proliferator-activated receptor gamma coactivator 1-alpha (Pgc-1α) axis. Pgc-1α stimulates the expression of nuclear respiratory factor (NRF), which leads to expression of mitochondrial transcription factor A (TFAM) and mitochondrial biogenesis. Pgc-1α stimulates and promotes mitochondrial biogenesis and capillary density as well as exercise capacity [10, 17, 18]. Consequently, aerobic exercise, also could increase *PGC-1*α expression [19]. Therefore it seems that there is a positive feedback on exercise capacity by PGC-1α.

Another key factor for promotion of mitochondrial biogenesis, oxidative capacity and preventing of the mitochondrial dysfunction in skeletal muscle is Sirt1 [20].

Also, up-regulation of mitochondrial biogenesis and increase in PGC-1α expression have been shown in a previous study [10]. Moreover, Aydin et al. indicated cardiac muscle produces more irisin than skeletal muscle [21]. Furthermore, BCAAs supplementation has been indicated to lead an increment of performance in rats subjected to moderate-intensity training [3]. Thus, the aim of present study is determination of BCAAs supplementation effects in association with aerobic training on plasma irisin and expression of gastrocnemius muscle *Fndc5*, *PPARγ*, *Pgc-1α*, *Sirt1* in male C57BL/6 mice.

## Methods

### Bioinformatics studies

A list of BCAAs and FNDC5 related proteins were extracted by an intensive literature review and text mining from a period of 2013–2017 (list is not shown). This list was subjected to the STRING V10.5 (https://string-db.org/) web site [22].In order to obtain the maximal data of FNDC5 and related proteins network, a low confidence level (> 0.150) was set. Among the visual network, which was obtained, PPARγ, Pgc-1α, Sirt1 were shown to be interacted with FNDC5 straightly. At the next step, STRING default confidential level was set to > 0.400 (mid confidence level) in order to define the interactions of PPARγ, Pgc-1α, Sirt1 with other proteins. We also assessed similarity of studied protein network between human and mouse via STRING V10.5.

### Ethical issue

Approval to perform experiments on mice was obtained by the ethics committee of Royan Institute (IR.ACECR.ROYAN.REC.1396.19).

### Animals and protocols

**Fig. 1:**
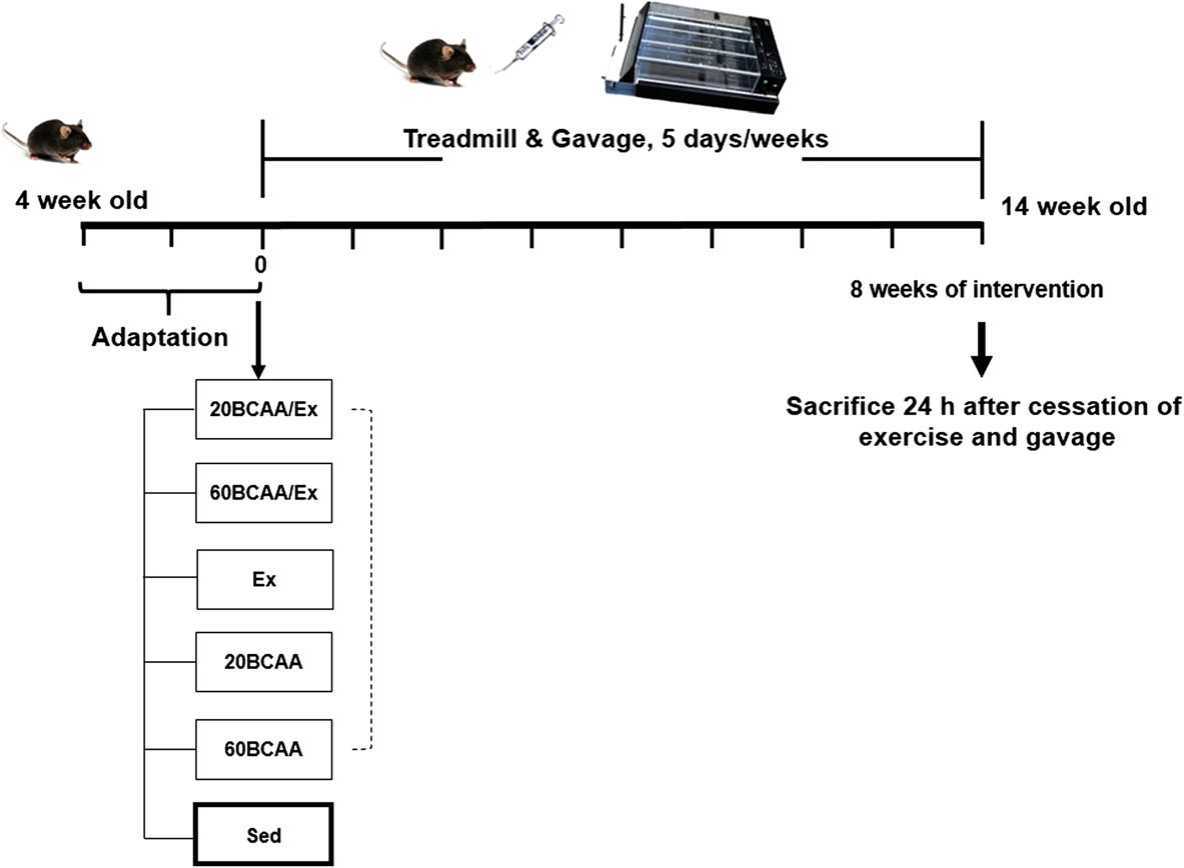
Schematic representataion of protocol used for BCAAs supplementation and exercise. As depicted, acclimation was performed for two weeks. Then, mice were randomly divided to training (*n* = 6) and sedantry (Sed; n = 6) groups. Training groups were EX as exercised group. 20BCAA and 60BCAA groups were mice supplemented with 20 and 60 mg/mL of BCAAs respectively. 20BCAA/EX and 60BCAA/EX were exercised groups supplemented with 20 and 60 mg/mL of BCAAs respectively. BCAAs supplementaion was performed by oral gavage (500 μL) 5 days per week. On the other hand mice were excercised by placing on the motor-driven treadmill as described in materials and methods for 5 days per week. Exercise and BCAAs supplemention was carried out as an intervention for 8 weeks. One day after the final session of intervention (gavage or exercise), mice were sacrified for further experiments

Four-week old wild type male C57BL/6 mice, were provided by Royan Institute (Tehran, Iran). Mice were housed in a temperature controlled room (24 ± 3 °C) with a humidity of 65% (±5%) and 12 h light/12 h dark cycle (lights from 08:00 am to 8:00 pm). Mice were allowed to acclimate for two weeks prior to the start of the experiment. Mice at their 6th weeks of age with an approximate weight of 18 ± 2 g, were randomly divided into six groups (*N* = 6). The groups were: (20BCAA/Ex): supplemented with 20 mg/mL of BCAAs with simultaneous exercise-training; (60BCAA/Ex): supplemented with 60 mg/mL of BCAAs with simultaneous exercise-training; (Ex): nonsupplemented, undergoing exercise; (20BCAA): supplemented with 20 mg/mL BCAAs; (60BCAA): supplemented with 60 mg/mL BCAAs (Sed): untrained mice without BCAAs intake (Fig. 1).

### BCAAs supplementation and food intake

Mice were fed with free access to standard food (13% (*w*/w) fat, 30% (w/w) protein, 57% (w/w) carbohydrate with a total 2900 kcal) and tap water. BCAAs (Bio nutrition, USA) were dissolved in distilled water and applied by gavage (500 μL) once per day for 8 weeks (20 mg/mL/day for 20BCAA group and 60 mg/mL/day for 60BCAA group). The reason for selecting 20 and 60 mg/mL of BCAAs was that 20 mg/mL of BCAAs was already indicated to contain beneficial effects [9, 23]. Therefore, 60 mg/mL of BCAAs (three times more concentrated dose) was considered as an overdose.

### Exercise training protocol, tolerance, and motor coordination tests

Endurance exercise training (Ex) and exhaustion tests were performed on a motorized treadmill. Ex was performed briefly as a type of moderate-high intensity exercise on treadmill for 8 weeks (5 days/week). After being acclimated to treadmill exercises over two weeks, running speed and duration of exercise were progressively increased to reach ~ 70% VO_2_ max (25 m/min). Whole exercise training was 45 min. The grade of treadmill slope was set to 0% and the speed of treadmill was set to increase in a rate of 3 m/min, initiating from 10 m/min to the final speed of 25 m/min (~ 70% VO_2_ max) [3, 19, 24]. Fixed speed rotarod was implemented to measure motor coordination and balance. Each mouse was received three trials per day for two consecutive days. The mice were trained at an initial constant speed of 8 rpm for 5 min. Then the speed was increased to 30 rpm for 5 min and the latency to fall was recorded. Time score was measured as the mean of the best performances over the three trials at the second day [10]. After 8 weeks of exercise and oral consumption of BCAAs, mice were fasted for 7 h before euthanasia. Mice were euthanized under the combined administration of xylazine (10 mg/kg body weight per mouse) and ketamine (80 mg/kg body weight per mouse) (Fig. 1).

### Quantitative real-time PCR (qRT-PCR)

Total RNA was extracted from gastrocnemius muscle and heart using TRIzol reagent (Sigma, USA). Samples were treated with DNaseI (TaKaRa) to remove contaminating genomic DNA. cDNA synthesis was performed with 1 μg of total RNA by cDNA synthesis kit according to the manufacturer’s instruction (TaKaRa). qRT-PCR was carried out with CYBR green (TaKaRa, Japan) using Corbet rotor gene 6000 (Qiagen, Australia). Assessment of gene expression was performed according to ΔΔCT method. Expression level of genes was reported relative to *glyceraldehyde-3-phosphate dehydrogenase* (*Gapdh*) expression level as already was implemented in similar studies [25–27].

Primers were ordered from micro-gene (South Korea) and their sequences are listed in Table 1.

**Table 1:** Primer list

Gene	Forward primer (5’-3’)	Reverse primer (5’-3’)	Annealing temperature (°C)	Accession no.
*Gapdh*	TGCCGCCTGGAGAAACC	TGAAGTCGCAGGAGACAACC	58.6	NM_008084.2
*Pgc-1α*	CCCTGCCATTGTTAAGACC	TGCTGCTGTTCCTGTTTTC	52	NM_008904.2
*Fndc5*	TCATTGTTGTGGTCCTCTTC	GCTCGTTGTCCTTGATGATA	60	NM_027402.3
*PPARγ*	TGAGACCAACAGCCTGAC	GTTCACCGCTTCTTTCAAATC	58	NM_001127330.1
*ATP5a1*	TCCGCTTACATTCCAACA	ACACAGACAAACCCACAT	54	NM_007505.2
*ATP5b*	GGTAGCGTTGGTATATGG	CTCCTGGTCTCTGAAGTA	54	NM_016774.3
*Cox4i1*	ATGGGAGTGTTGTGAAGAGT	CATCAGGCAAGGGGTAGT	54	NM_009941.3
*Tfam*	CTTCAACCACCACACCACT	AATCTCTAAGCCTCCTCAATACAA	56	NM_009360.4
*Sirt1*	GGCAGTAACAGTGACAGT	CTCTCCGTATCATCTTCCAA	61	NM_001159589.2

### Protein extraction and western blotting

Tissues were lysed using TRI reagent (Thermo Scientific, 15,596–018), according to the manufacturer protocol. Equal amounts of each protein sample (30 μg) were separated by SDS- PAGE and transferred to PVDF membranes (Bio Rad, 162–0176). After blocking the membranes with 10% skim milk (Millipore, 115,363), membranes were incubated withdifferent primary antibodies for 2 h at room temperature. Primary antibodies were rabbit anti-FNDC5 antibody (1:2000, Abcam, AB174833) and mouse anti GAPDH antibody (1:5000, Santa Cruz). Then, membranes were incubated for 1 h at room temperaturewith an appropriate secondary antibody: horseradish peroxidase (HRP)-conjugated goat antimouse IgG (1:5000, Dako, P0447), or HRP-conjugated goat anti-rabbit IgG (1:16000, Santa Cruz, SC2301). HRP-conjugated IgG bound to each protein band was visualized by anAmersham ECL Advance Western Blotting Detection Kit (GE Healthcare). The intensity of each band was quantified by Image J software.

### Plasma measurement of irisin, urea and lactate

Blood was collected from the right ventricle of each mouse and drawn into an EDTA containing tube. To separate plasma, blood containing tubes were centrifuged for 15 min at 1600×g at 4 °C. All samples were frozen in liquid nitrogen and stored at − 80 °C until further use. Plasma irisin levels were measured using a commercially available enzyme-linked immunosorbent assay ELISA Kit (EK-067-29, Phoenix, USA). The assay kit was highly sensitive to irisin in mice and detection range of this kit was 0.1–1000 ng/mL. Meanwhile, plasma urea concentration (Pars Azmon, Iran) and lactate (Biorex Fars, Iran) were measured according to the manufacturer’s instructions.

### Statistical analysis

Statistical analysis was performed using GraphPad Prism Software (Version 7.0a Graph Pad Software Inc., La Jolla, CA). Kolmogorov–Smirnov test was used for normalizing of distribution, and variables were normally distributed. Results are presented as mean ± standard error of mean (SEM). Data of RT-qPCR and western blotting were obtained on triplicate data sets for each sample and analyzed by two-way analysis of variance (ANOVA) with tukey’s post hoc test due to multiple comparisons. Differences at *p* < 0.05 were considered to be significant in all analyses.

## Results

### Bioinformatics analyses indicated a connection ring of FNDC5 with SIRT1, PPARγ, Pgc-1α, and TFAM

**Fig. 2:**
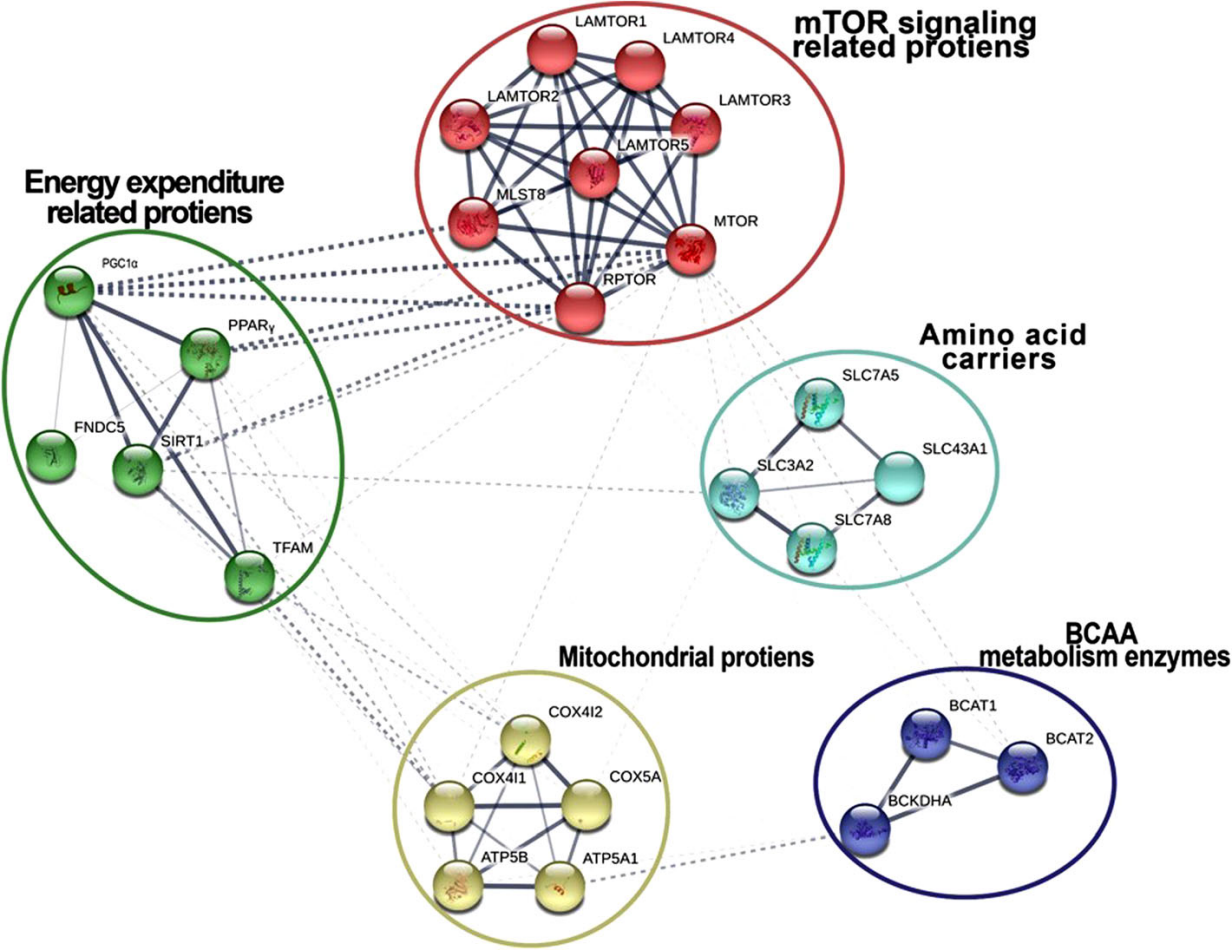
STRING analysis to unravel interactions between FNDC5 with SIRT1, PPARγ, Pgc-1α, and TFAM proteins in human. As detailed in materials and methods, low-confidence interactions in STRING (string-db.org) indicated interaction among SIRT1, PPARγ, Pgc-1α, and TFAM proteins in mouse. TFAM and its downstream proteins including COX4I1, COX4I2, COX5A, ATP5A1, and ATP5B are shown. Also there were strong interactions with SIRT1, PPARγ, Pgc-1α and mTOR signaling components as evidenced that mTOR components are regulated by BCAAs. Moreover, there is an evidence for interaction between proteins involved in BCAAs metabolism and ATP5A1 (a mitochondrial protein). This schematic representation evidences that there is a coordination between energy expenditure proteins and mTOR signaling components as well as amino acid carriers, mitochondrial proteins and enzymes in BCAAs metabolism

STRING low confidence level showed a text mining-based interaction between FNDC5 with SIRT1, PPARγ, Pgc-1α, and TFAM (Fig. 2). Next, analyses of high-confidence interactions indicated a strong interaction among SIRT1, PPARγ, Pgc-1α, and TFAM proteins (Data not shown). As TFAM is in connection with mitochondrial DNA (mtDNA) replication and transcription activity, its downstream proteins including COX4I1, COX4I2, COX5A, ATP5A1 and ATP5B are also involved. Of important, there is a high similarity in protein sequences between human and mouse for a number of aforementioned proteins emphasizing similar text mining-based interactions between these proteins in human (Additional file 1: Figure S1) [22].

### Animal characteristics measurements

**Fig. 3:**
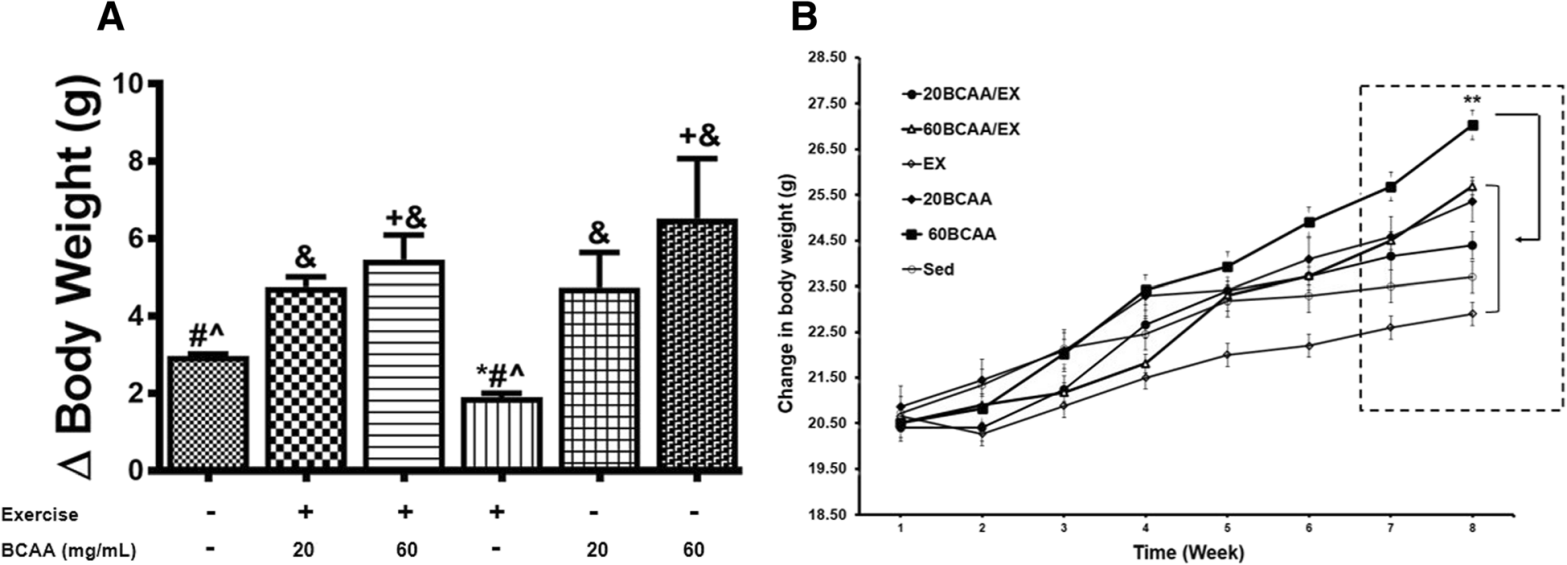
Modulation in body weight of exercised and BCAAs supplemented mice. Mice were weighed every weeks and an average for each group (*n* = 6) was recorded weekly (**b**). On the other hand, weight gain for each mouse was calculated by subtracting the weight at first week from the weight at 8th week (**a**). Sed indicates no received BCAAs and sedentary mice. Ex stands for exercised group. 20BCAA and 60BCAA groups are mice supplemented with 20 and 60 mg/mL of BCAAs respectively. 20BCAA/EX and 60BCAA/EX are exercised groups supplemented with 20 and 60 mg/mL of BCAAs respectively. As depicted the weight gain was maximal in 60BCAA group (**b**). Data are shown as mean ± SEM (Standard Error of Mean). ** indicates significant difference with Sed group at level of *p* < 0.01 at quadrant. Data were analyzed by two-way analysis of variance (ANOVA) and tukey’s post hoc test

**Fig. 4:**
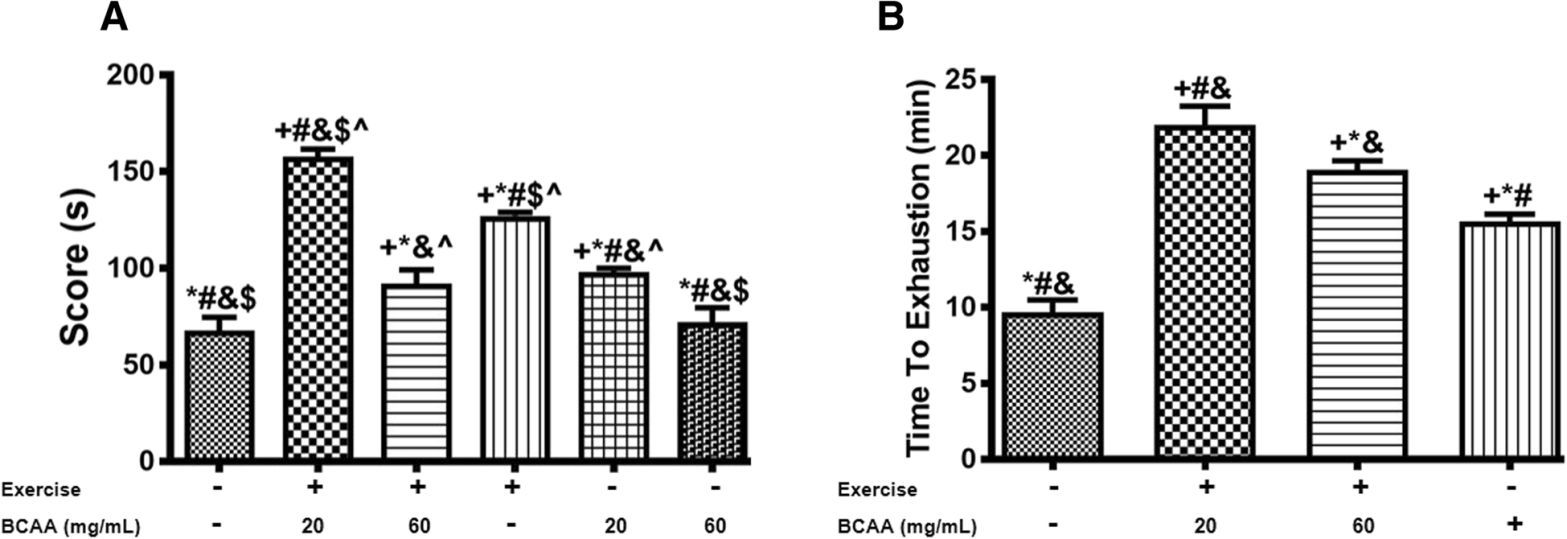
Endurance capacity and coordination function in treated groups compare with Sed group. (**a**) Rotarod performance was applied for each group (*n* = 6) and the mean of score for each group is shown as mean ± SEM (Standard Error of Mean). Exercise performance and BCAAs supplementation significantly improved rotarod performance (*p* < 0.05). However simultaneous application of performance and BCAAs supplementation did not exert additive effect on performance. S stands for second. (**b**) Time to reach exhaustion was measured in trained groups vs sedentary control group. Of important, exercised group supplemented with 20BCAA reached to maximal endurance capacity. + indicates statistically significant difference with Sed group at *p* < 0.05, * indicates statistically significant difference with 20BCAA/Ex group at *p* < 0.05, # indicates statistically significant difference with 60BCAA/Ex group at *p* < 0.05, & indicates statistically significant difference with Ex group at *p* < 0.05, $ indicates statistically significant difference with 20BCAA group at *p* < 0.05, ^ indicates statistically significant difference with 60BCAA group at *p* < 0.05. Data were analyzed by two-way analysis of variance (ANOVA) and tukey’s post hoc test

Body weight (Fig. 3a, b) and gastrocnemius muscle weight (Table 2) were significantly increased in 60BCAA group. Upon eight weeks performance of tolerance and motor coordination test, 20BCAA/Ex group, got the highest score (Fig. 4a). Also by taking exhausting test, we indicated no improve in endurance capacity for 60BCAA/Ex group compared with 20BCAA/Ex group (Fig. 4b).

**Table 2:** Heart and gastrocnemius muscle weight of mice

Groups		Heart Weight (g)	Gastrocnemius Weight (g)
BCAAs (mg/mL)	Exercise		
–	–	0.14 ± 0.01^*#&^	0.15 ± 0.01^&$^^
20	+	0.20 ± 0.02^+$^^	0.16 ± 0.01^$^^
60	+	0.22 ± 0.03^+&$^^	0.17 ± 0.26^$^^
–	+	0.18 ± 0.02^+#^	0.18 ± 0.01^+^^
20	–	0.15 ± 0.01^*#^	0.21 ± 0.01^+*#^
60	–	0.15 ± 0.01^*#^	0.23 ± 0.02^+*#&^

Values are expressed as mean ± SEM. + indicates statistically significant difference with Sedentary group at *p* < 0.05, * indicates statistically significant difference with 20BCAA/Ex group at *p* < 0.05, # indicates statistically significant difference with 60BCAA/Ex group at *p* < 0.05, & indicates statistically significant difference with Ex group at *p* < 0.05, $ indicates statistically significant difference with 20BCAA group at *p* < 0.05, ^ indicates statistically significant difference with 60BCAA group at *p* < 0.05

### Effect of different concentrations of BCAA and aerobic exercise on FNDC5

**Fig. 5:**
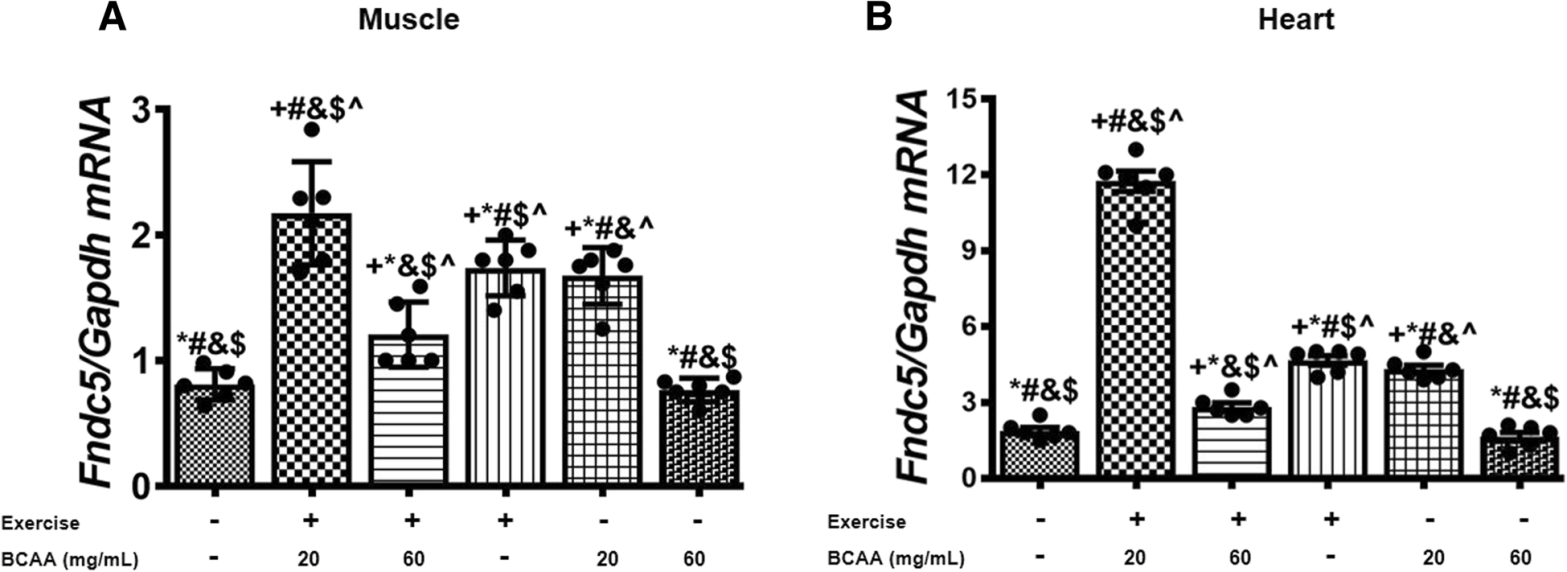
*Fndc5* transcript levels were increased in heart and muscle tissues of exercised and BCAAs supplemented mice. As elucidated, qRT-PCR on cDNA samples of gastrocnemius muscle (**a**) and heart (**b**) tissues derived from exercised and BCAAs supplemented mice indicated a significant increase in *Fndc5* transcript level (*p* < 0.05). Data are shown as mean ± SEM (Standard Error of Mean) for each group (*n* = 6, ●). + indicates statistically significant difference with Sed group at *p* < 0.05, * indicates statistically significant difference with 20BCAA/Ex group at *p* < 0.05, # indicates statistically significant difference with 60BCAA/Ex group at *p* < 0.05, & indicates statistically significant difference with Ex group at *p* < 0.05, $ indicates statistically significant difference with 20BCAA group at *p* < 0.05, ^ indicates statistically significant difference with 60BCAA group at *p* < 0.05. Data of qRT-PCR were obtained on triplicate data sets for each sample and analyzed by two-way analysis of variance (ANOVA) and tukey’s post hoc test

**Fig. 6:**
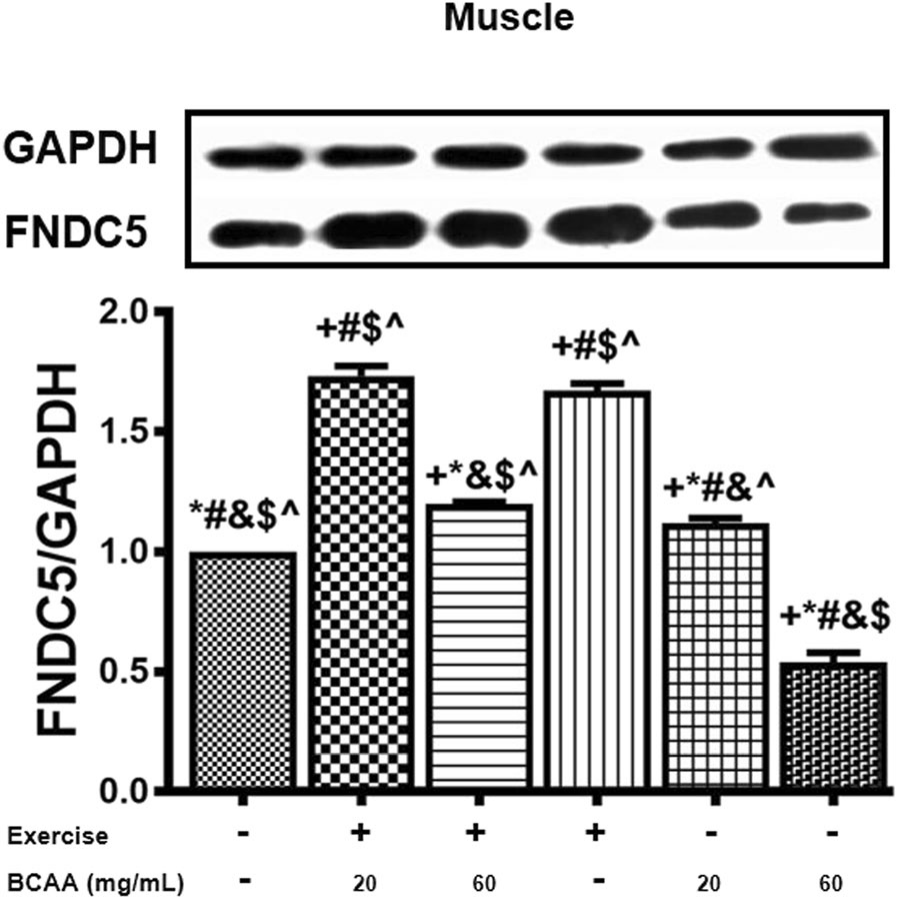
Fndc5 protein contents were increased in gastrocnemius muscle tissue of exercised and BCAAs supplemented mice. Similar to what observed for the *Fndc5* transcript levels, protein contents of FNDC5 was highest in 20BCAA/Ex group at *p* < 0.05 as well as Ex group. Data are shown as mean ± SEM (Standard Error of Mean) for each group (*n* = 6, ●). For better comparison, one of the immunoblots is shown above the diagram. + indicates statistically significant difference with Sed group at *p* < 0.05, * indicates statistically significant difference with 20BCAA/Ex group at *p* < 0.05, # indicates statistically significant difference with 60BCAA/Ex group at *p* < 0.05, & indicates statistically significant difference with Ex group at *p* < 0.05, $ indicates statistically significant difference with 20BCAA group at *p* < 0.05, ^ indicates statistically significant difference with 60BCAA group at *p* < 0.05. Data of western blotting were obtained on triplicate data sets for each sample and the intensity of each bond was quantified by image J. Two-way analysis of variance (ANOVA) and tukey’s post hoc test were used for statistical analysis

Data revealed that *Fndc5* expression was significantly increased in both, 20BCAA/Ex, and 60 BCAA/Ex comparing to Sed group (untrained without BCAAs intake -) in heart and gastrocnemius muscles (Fig. 5a, b). Interestingly, supplementation with 60 mg/mL BCAAs was not able to increase *Fndc5* expression, while at lower concentration of BCAA (20 mg/mL) upregulated the expression of *Fndc5* significantly (Fig. 5a, b). Furthermore, the highest increase in *Fndc5* expression was accomplished when a combination of exercise was performed with 20 mg/mL BCAAs supplementation (20BCAA/Ex group) (Fig. 5a, b). In the gastrocnemius skeletal muscle, protein levels of FNDC5 were significantly higher in Ex and 20BCAA/Ex groups as compared to Sed group. Similar to the *Fndc5* mRNA, protein content of FNDC5 was not up-regulated in 60BCAA group (Fig. 6).

### BCAAs supplementation and aerobic exercise effects on the expression of genes involved in mitochondrial biogenesis

**Fig. 7:**
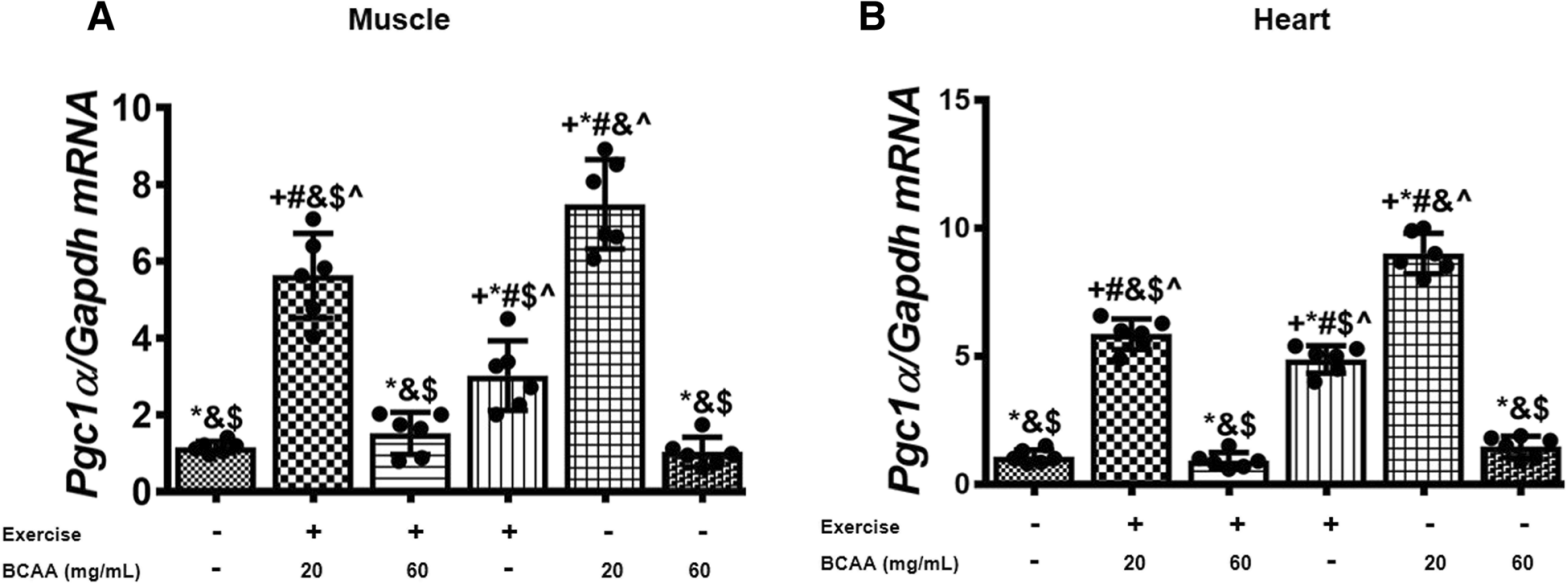
Modulation in transcript levels of *Tfam* in muscle and heart tissues of exercised and BCAAs supplemented mice. qRT-PCR on cDNA samples of gastrocnemius muscle (**a**) and heart (**b**) tissues derived from exercised and BCAAs supplemented mice indicated a most increase of *Tfam* transcript level in 20BCAA group (*p* < 0.05). Data are shown as mean ± SEM (Standard Error of Mean) for each group (*n* = 6, ●). + indicates statistically significant difference with Sedentary group at *p* < 0.05, * indicates statistically significant difference with 20BCAA/Ex group at *p* < 0.05, # indicates statistically significant difference with 60BCAA/Ex group at *p* < 0.05, & indicates statistically significant difference with Ex group at *p* < 0.05, $ indicates statistically significant difference with 20BCAA group at *p* < 0.05, ^ indicates statistically significant difference with 60BCAA group at *p* < 0.05. Data of qRT-PCR were obtained on triplicate data sets for each sample and analyzed by two-way analysis of variance (ANOVA) and tukey’s post hoc test

**Fig. 8:**
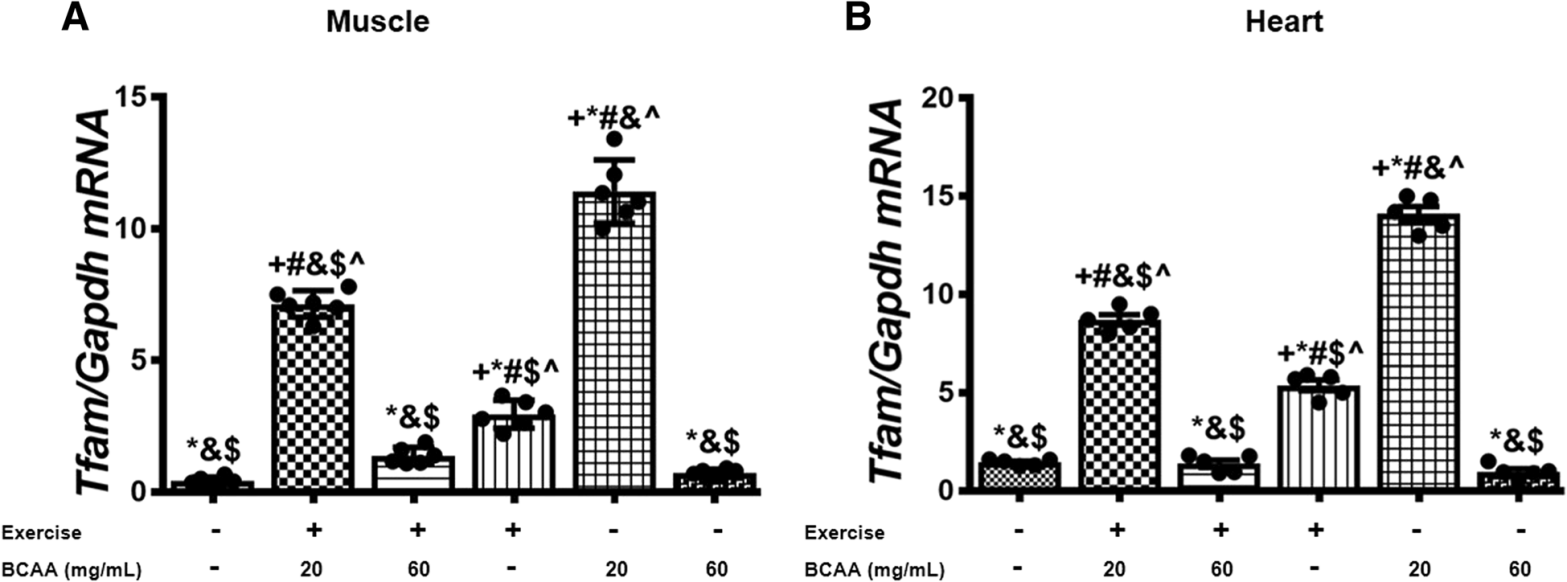
Variation in relative expression levels of *Cox4i1* in muscle and heart tissues of exercised and BCAAs supplemented mice. qRT-PCR on cDNA samples of gastrocnemius muscle (**a**) and heart (**b**) tissues derived from exercised and BCAAs supplemented mice indicated a significant increase of *Cox4i1* transcript level in in 20BCAA group (*p* < 0.05). Data are shown as mean ± SEM (Standard Error of Mean) for each group (*n* = 6, ●). + indicates statistically significant difference with Sedentary group at *p* < 0.05, * indicates statistically significant difference with 20BCAA/Ex group at *p* < 0.05, # indicates statistically significant difference with 60BCAA/Ex group at *p* < 0.05, & indicates statistically significant difference with Ex group at *p* < 0.05, $ indicates statistically significant difference with 20BCAA group at *p* < 0.05, ^ indicates statistically significant difference with 60BCAA group at *p* < 0.05. Data of qRT-PCR were obtained on triplicate data sets for each sample and analyzed by two-way analysis of variance (ANOVA) and tukey’s post hoc test

**Fig. 9:**
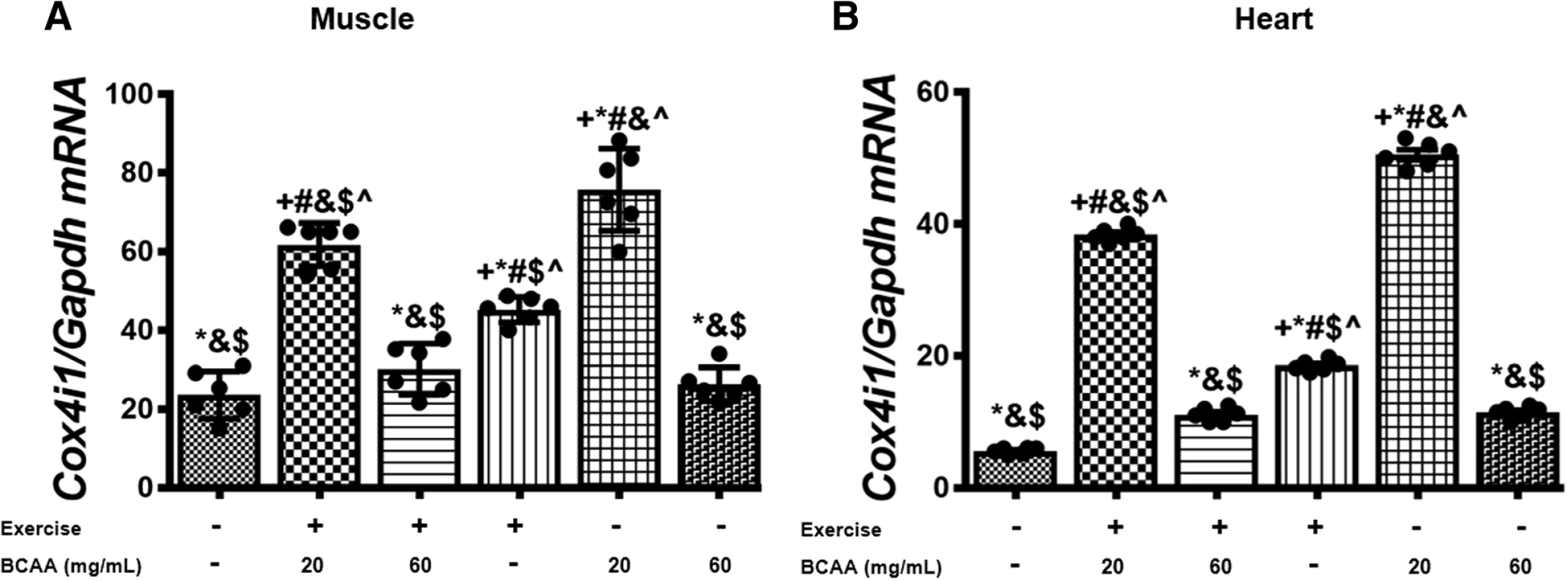
Modulation in relative expression levels of *ATP5a1* in muscle and heart tissues of exercised and BCAAs supplemented mice. qRT-PCR on cDNA samples of gastrocnemius muscle (**a**) and heart (**b**) tissues derived from exercised and BCAAs supplemented mice indicated a significant increase of *ATP5a1* transcript level in 20BCAA group (*p* < 0.05). Data are shown as mean ± SEM (Standard Error of Mean) for each group (*n* = 6, ●). + indicates statistically significant difference with Sedentary group at *p* < 0.05, * indicates statistically significant difference with 20BCAA/Ex group at *p* < 0.05, # indicates statistically significant difference with 60BCAA/Ex group at *p* < 0.05, & indicates statistically significant difference with Ex group at *p* < 0.05, $ indicates statistically significant difference with 20BCAA group at *p* < 0.05, ^ indicates statistically significant difference with 60BCAA group at *p* < 0.05. Data of qRT-PCR were obtained on triplicate data sets for each sample and analyzed by two-way analysis of variance (ANOVA) and tukey’s post hoc test

**Fig. 10:**
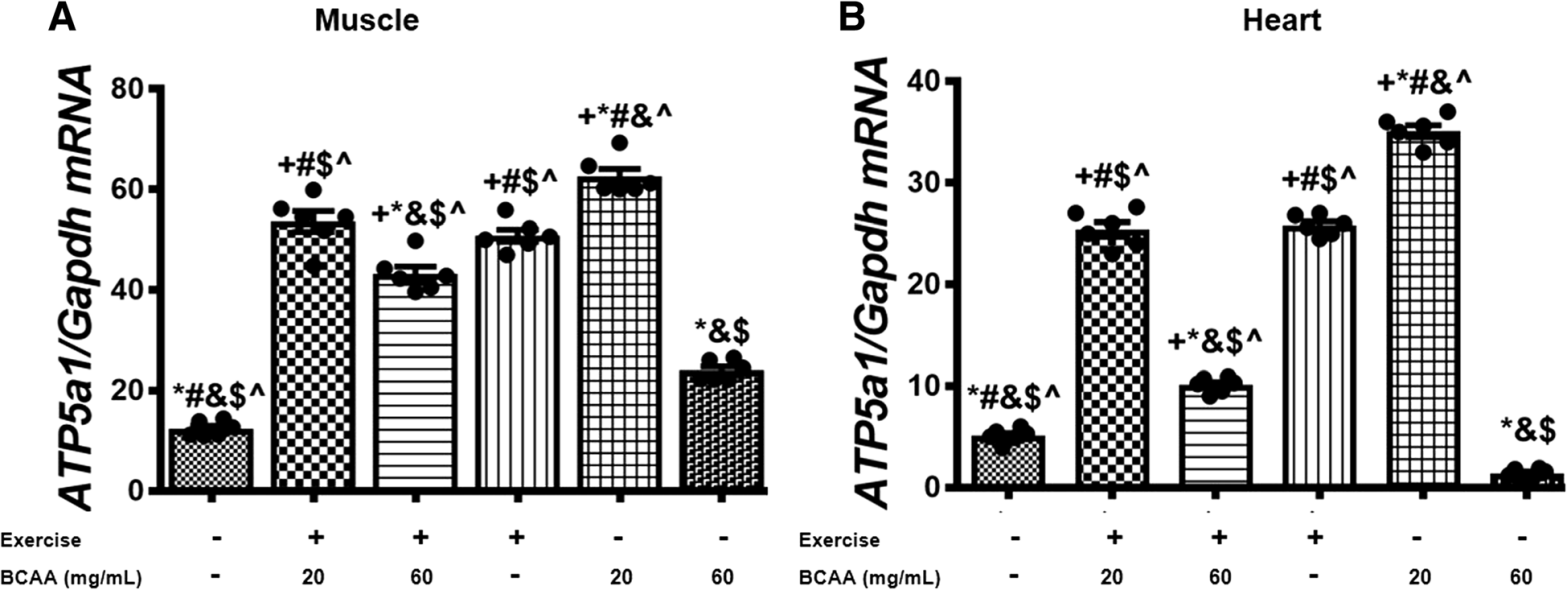
Variation in expression level of *ATP5b* in muscle and heart tissues of exercised and BCAAs supplemented mice. qRT-PCR on cDNA samples of gastrocnemius muscle (**a**) and heart (**b**) tissues derived from exercised and BCAAs supplemented mice indicated a significant increase of *ATP5b* transcript level in 20BCAA group (*p* < 0.05). Data are shown as mean ± SEM (Standard Error of Mean) for each group (*n* = 6, ●). + indicates statistically significant difference with Sedentary group at *p* < 0.05, * indicates statistically significant difference with 20BCAA/Ex group at *p* < 0.05, # indicates statistically significant difference with 60BCAA/Ex group at *p* < 0.05, & indicates statistically significant difference with Ex group at *p* < 0.05, $ indicates statistically significant difference with 20BCAA group at *p* < 0.05, ^ indicates statistically significant difference with 60BCAA group at *p* < 0.05. Data of qRT-PCR were obtained on triplicate data sets for each sample and analyzed by two-way analysis of variance (ANOVA) and tukey’s post hoc test

**Fig. 11:**
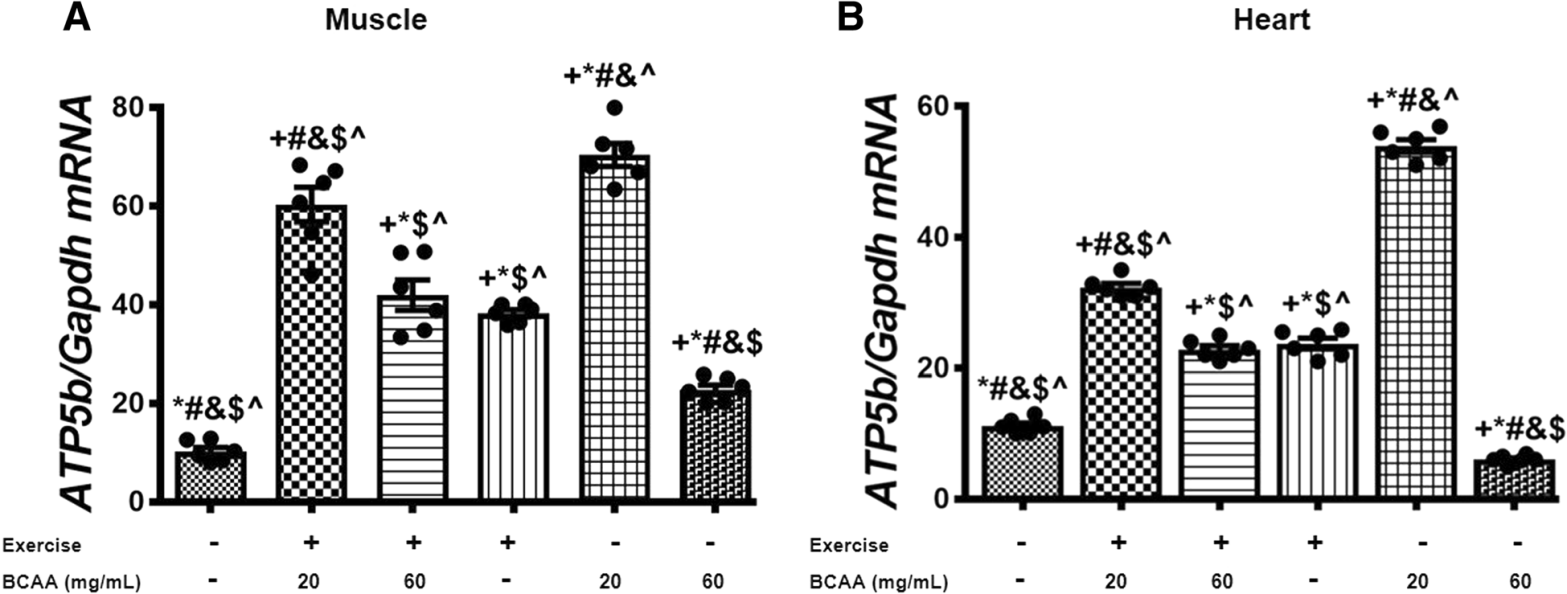
Expression levels of Pgc-1α was increased in heart and muscle tissues of exercised and 20 mg/mL BCAAs (20BCAA/Ex) supplemented mice. qRT-PCR on cDNA samples of gastrocnemius muscle (**a**) and heart (**b**) tissues derived from exercised and BCAA supplemented mice indicated most increase of Pgc-1α transcript level in 20BCAA group (p < 0.05). Data are shown as mean ± SEM (Standard Error of Mean) for each group (n = 6, ●). + indicates statistically significant difference with Sedentary group at p < 0.05, * indicates statistically significant difference with 20BCAA/Ex group at p < 0.05, # indicates statistically significant difference with 60BCAA/Ex group at p < 0.05, & indicates statistically significant difference with Ex group at p < 0.05, $ indicates statistically significant difference with 20BCAA group at p < 0.05, ^ indicates statistically significant difference with 60BCAA group at p < 0.05. Data of qRT-PCR were obtained on triplicate data sets for each sample and analyzed by two-way analysis of variance (ANOVA) and tukey’s post hoc test

**Fig. 12:**
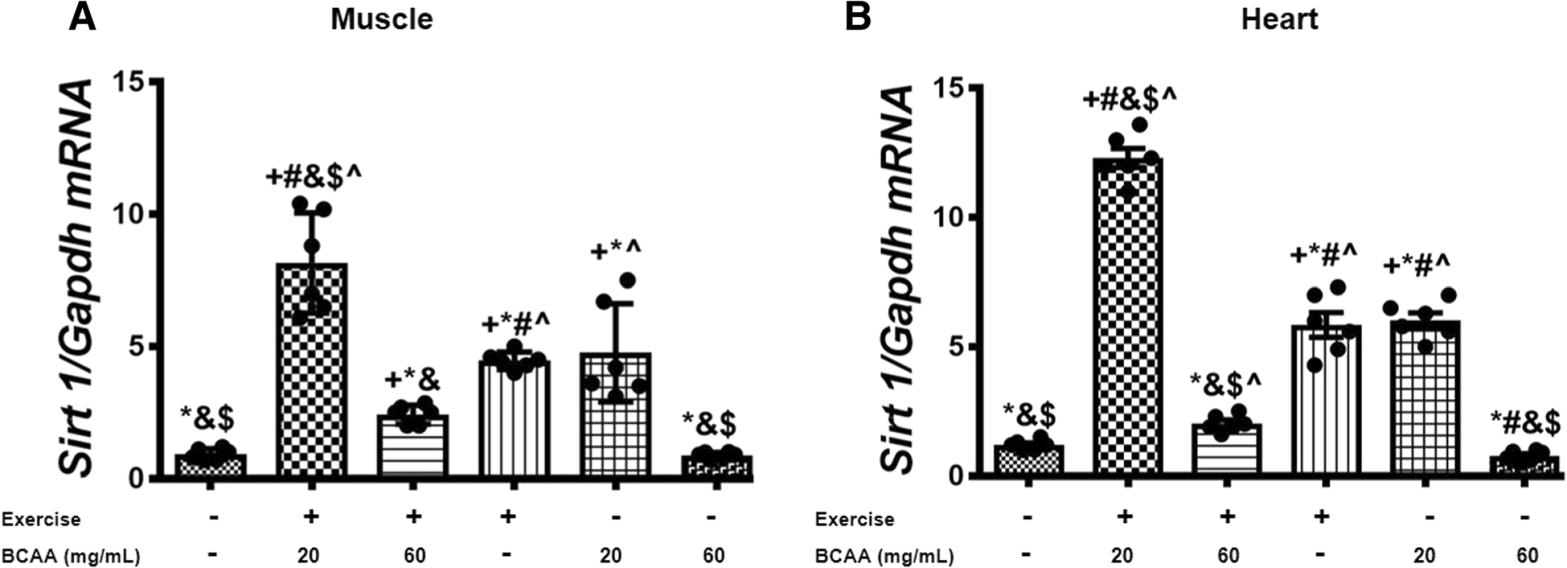
Modulation in relative expression level of *Sirt1* in gastrocnemius and heart muscles of exercised and BCAAs supplemented mice. qRT-PCR on cDNA samples of gastrocnemius muscle (**a**) and heart (**b**) tissues derived from exercised and 20 mg/mL BCAAs supplemented mice (20BCAA/Ex) indicated a significant increase of *Sirt1* transcript level (*p* < 0.05). Data are shown as mean ± SEM (Standard Error of Mean) for each group (*n* = 6, ●). + indicates statistically significant difference with Sedentary group at *p* < 0.05, * indicates statistically significant difference with 20BCAA/Ex group at *p* < 0.05, # indicates statistically significant difference with 60BCAA/Ex group at *p* < 0.05, & indicates statistically significant difference with Ex group at *p* < 0.05, $ indicates statistically significant difference with 20BCAA group at *p* < 0.05, ^ indicates statistically significant difference with 60BCAA group at *p* < 0.05. Data of qRT-PCR were obtained on triplicate data sets for each sample and analyzed by two-way analysis of variance (ANOVA) and tukey’s post hoc test

**Fig. 13:**
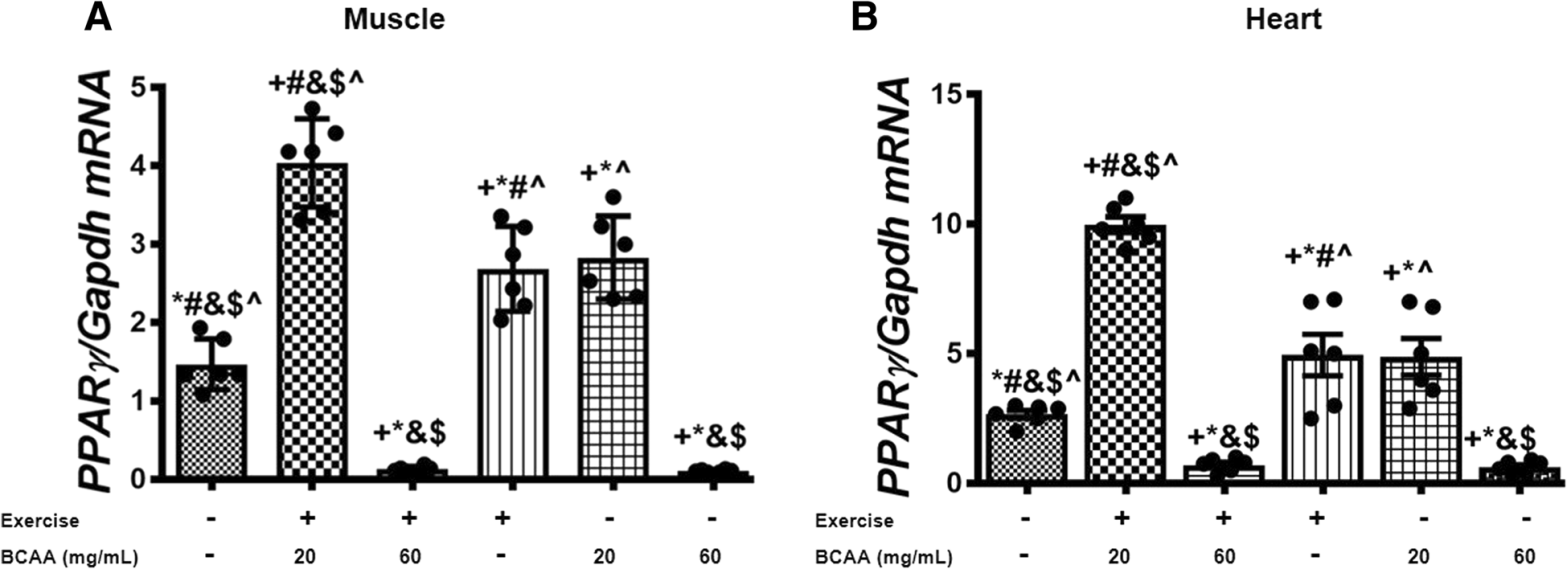
Modulation in transcript level of *PPARγ* in gastrocnemius muscle and heart tissues of exercised and BCAAs supplemented mice. qRT-PCR on cDNA samples of gastrocnemius muscle (**a**) and heart (**b**) tissues derived from exercised and 20 mg/mL BCAAs supplemented mice (20BCAA/Ex) indicated a significant increase in *PPARγ* transcript level (*p* < 0.05). Interestingly a repression in expression of *PPARγ* was observed with BCAAs supplementation (60 mg/mL) [60BCAA]. Data are shown as mean ± SEM (Standard Error of Mean) for each group (n = 6, ●). + indicates statistically significant difference with Sedentary group at *p* < 0.05, * indicates statistically significant difference with 20BCAA/Ex group at *p* < 0.05, # indicates statistically significant difference with 60BCAA/Ex group at *p* < 0.05, & indicates statistically significant difference with Ex group at *p* < 0.05, $ indicates statistically significant difference with 20BCAA group at *p* < 0.05, ^ indicates statistically significant difference with 60BCAA group at *p* < 0.05. Data of qRT-PCR were obtained on triplicate data sets for each sample and analyzed by two-way analysis of variance (ANOVA) and tukey’s post hoc test

Additionally, we assessed the expression of mitochondrial genes in gastrocnemius and heart muscles. As expected, our data indicated that BCAAs increased the expression of mtDNA transcription factor A (*Tfam*), *Cox4i1*, a and b subunits of the mitochondrial H^+^-ATP synthase (*a-F1-ATPase, b-F1-ATPase*) in both of gastrocnemius and heart muscles (Figs. 7, 8, 9, 10). However, the maximum enhancement was yielded when BCAAs at concentration of 20 mg/mL (20BCAA) was supplemented. Of important the effect of exercise was not significant with BCAAs supplementation (Figs. 7, 8, 9, 10). A similar trend of modulation in transcripts levels of aforementioned genes was obtained in heart tissue of the mice (Figs. 7, 8, 9, 10). As these genes are under regulation of Pgc-1α, we assessed transcript level of *Pgc-1α* in both gastrocnemius and heart tissues. Again, the same trend was observed for *Pgc-1α* expression in both heart and muscle tissues (Fig. 11). Exercise (Ex) increased transcript levels of *Pgc-1α* (Fig. 11), whereas maximal increase in *Pgc-1α* mRNA was yielded when 20 mg/mL BCAAs supplemented (20BCAA) (Fig. 11). Importantly, no significant modulation in *Pgc-1α* transcript levels was observed when higher amount of BCAAs (60 mg/mL) [60BCAA] was implemented (Fig. 11). Data revealed that increased expression of *Pgc-1α* due to the administration of 20 mg/mL BCAAs (20BCAA) was more than exercise (Ex) (Fig. 11). Notably, we found that *Sirt1* mRNA was increased in 20BCAA/Ex group, the same as *Fndc5* mRNA in gastrocnemius and heart muscle (Fig. 12). To our knowledge, *Pgc-1α* is known as the co-activator of *PPARγ*. Data indicated that *PPARγ* gene expression was significantly increased in exercise group (Ex) (Fig. 13). Interestingly, administration of 60 mg/mL BCAAs during the exercise (60BCAA/Ex) decreased transcript levels of *PPARγ* (Fig. 13). However supplementation of 20 mg/mL BCAAs with exercise (20BCAA/Ex) significantly increased the expression of *PPARγ* (Fig. 13). Moreover, high amount of BCAAs (60 mg/mL) [60BCAA] administration reversed the expression of *PPARγ* (Fig. 13)*.*

### Correlation analysis of circulating irisin with muscle and heart *Fndc5* mRNAs

**Fig. 14:**
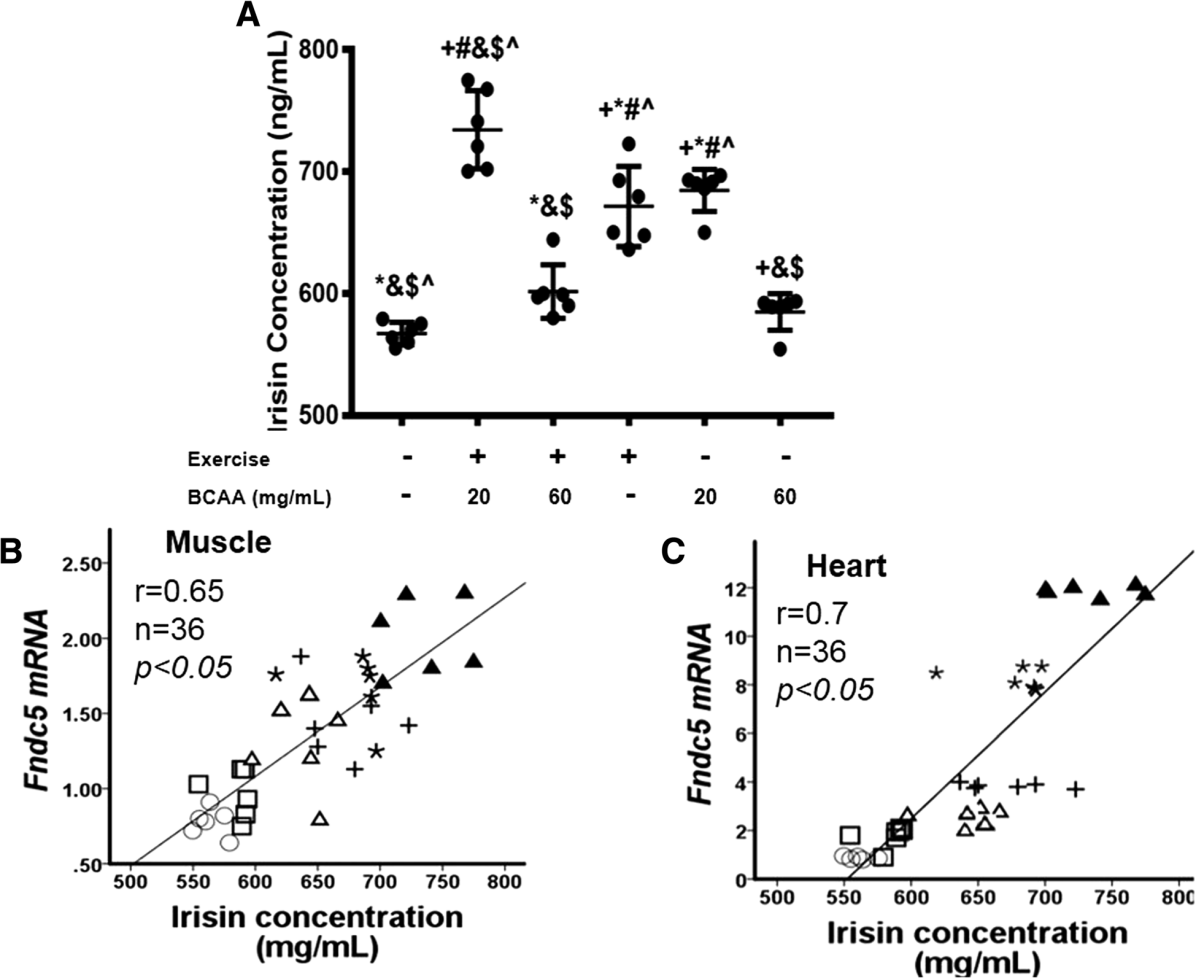
Measurement of plasma irisin and correlation analysis of irisin modulation with BCAAs supplementation and exercise. Irisin content was measured by ELISA assay on plasma samples (**a**). A synergistic effect of simultaneous exercise and BCAAs supplementation (20 mg/mL) [20BCAA/Ex] was obtained on irisin concentration. Data are shown as mean ± SEM (Standard Error of Mean) for each group (n = 6, ●). + indicates statistically significant difference with Sedentary group at *p* < 0.05, * indicates statistically significant difference with 20BCAA/Ex group at *p* < 0.05, # indicates statistically significant difference with 60BCAA/Ex group at *p* < 0.05, & indicates statistically significant difference with Ex group at *p* < 0.05, $ indicates statistically significant difference with 20BCAA group at *p* < 0.05, ^ indicates statistically significant difference with 60BCAA group at *p* < 0.05. Data of qRT-PCR were obtained on triplicate data sets for each sample and analyzed by two-way analysis of variance (ANOVA) and tukey’s post hoc test. Meanwhile, a correlation analysis performed between the plasma irsin and transcript levels of *Fndc5* of gastrocnemius muscle tissues (**b**) and heart muscle (**c**).○ Stands for the Sed group (no BCAAs supplementation, no exercise). + stands for exercise group (Ex group). * represents BCAAs supplemented group (20 mg/mL) [20BCAA].□ represents BCAAs supplemented group (60 mg/mL) [60BCAA]. Δ represents BCAAs supplemented (60 mg/mL) exercised group [60BCAA/Ex]. ▲ represents BCAAs supplemented exercised group (20 mg/mL) [20BCAA/Ex]. As depicted, most increase in irisin was observed in plasma of 20BCAA/Ex group which showed maximal increase in *Fndc5* transcript levels of heart and gastrocnemius muscle tissues

The impact of exercise on plasma levels of irisin was later investigated by ELISA. As depicted, ELISA results revealed that plasma concentration of irisin significantly was changed in mice 20BCAA/Ex and EX groups compared to Sed group (Fig. 14a)*.* Correlation of irisin levels with *Fndc5* revealed that circulating irisin had positive associations (*r* = 0.65 and 0.7 for muscle and heart respectably) with the expression of this gene (Fig. 14b, c). Interestingly, increased amount of plasma irisin was related to the increased expression level of *Fndc5* in 20BCAA/Ex group.

### Plasma profile of lactate and urea in exercised and BCAAs supplemented mice

**Fig. 15:**
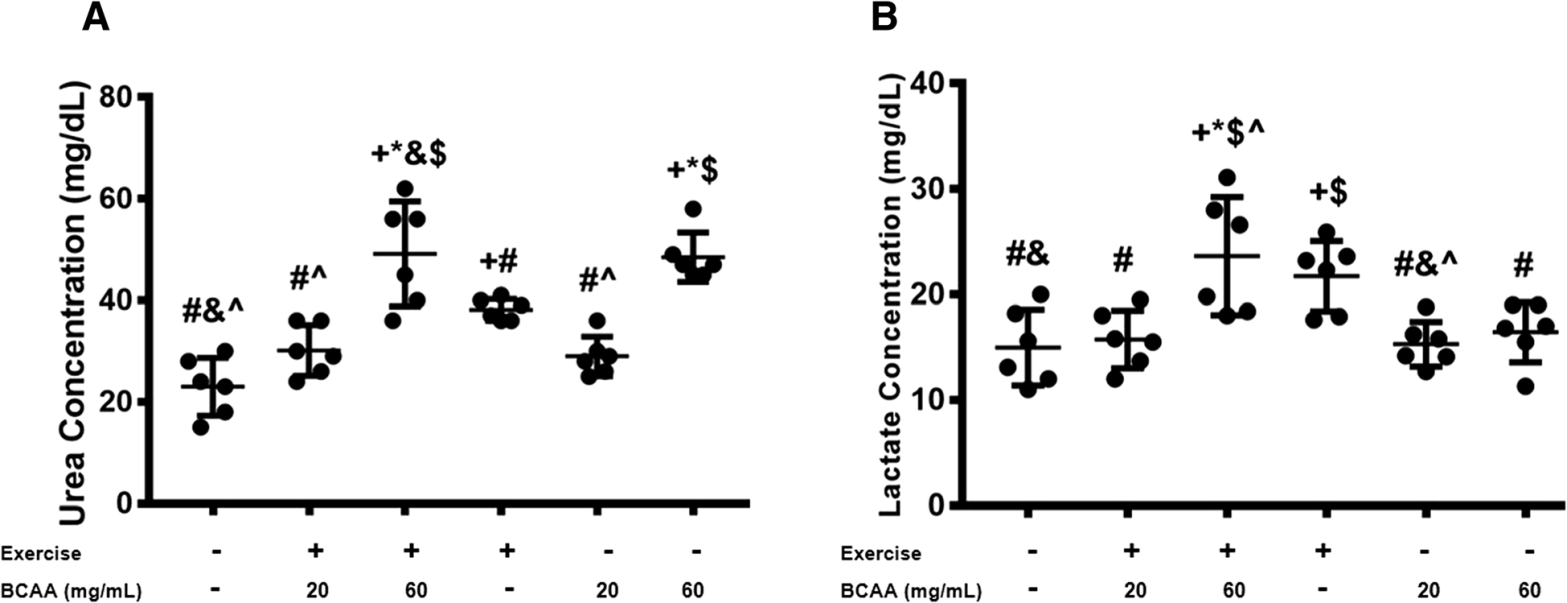
Measurement of plasma lactate and urea in exercised and BCAAs mice. Urea (**a**) and lactate (**b**) content was measured by calorimetric assays as elucidated in materials and methods. Maximal increase on content of both parameters were obtained in 60BCAA/Ex group. Data are shown as mean ± SEM (Standard Error of Mean) for each group (*n* = 6, ●). + indicates statistically significant difference with Sedentary group at *p* < 0.05, * indicates statistically significant difference with 20BCAA/Ex group at *p* < 0.05, # indicates statistically significant difference with 60BCAA/Ex group at *p* < 0.05, & indicates statistically significant difference with Ex group at *p* < 0.05, $ indicates statistically significant difference with 20BCAA group at *p* < 0.05, ^ indicates statistically significant difference with 60BCAA group at *p* < 0.05. Data of qRT-PCR were obtained on triplicate data sets for each sample and analyzed by two-way analysis of variance (ANOVA) and tukey’s post hoc test

Meanwhile, lactate and urea content in plasma increased significantly in 60BCAA/Ex group (Fig. 15)*.* Importantly, we did not observe any significant change in aforementioned parameters in 20BCAA/Ex group. Consistently, in 20BCAA/Ex group whole endurance capacity in treadmill tests was improved (Fig. 4b) as well as better performance on motor coordination test (Fig. 4a).

## Discussion

Our results indicated that BCAAs supplementation increased body weight, heart and gastrocnemius muscle weight. As it was earlier reported, BCAAs supplementation promotes protein synthesis and inhibits protein degradation via mTOR pathway, therefore increases muscle and heart weight [6] Also, importance of cardiac BCAAs catabolism and functional impact on cardiac development and physiology is well recognized [28]. D’Antona et al. indicated that BCAAs supplementation increases mitochondrial biogenesis through *Pgc-1α* and *Sirt1* expression in primary cardiac and skeletal myocytes, which is accompanied by enhanced physical endurance They also demonstrated BCAAs supplemented mice was accompanied by enhanced mitochondrial biogenesis and function in cardiac and skeletal muscles but not in liver or fat. They showed that BCAAs activated mTOR signaling and could enhance mitochondrial biogenesis [10].

Physical exercise is a well-recognized factor that could increase *Fndc5* gene expression [14, 21, 30]. Our results confirmed such concept as exercise increased transcript level of muscle and heart *Fndc5*. Interestingly BCAAs (20 mg/mL) supplementation (20BCAA) was beneficial to increase the transcript level of *Fndc5*. However, higher amount of BCAAs (60 mg/mL) did not change *Fndc5* transcript level even though the expression of mitochondrial genes decreased. This is possibly due to triggering an increase in energy expenditure [30]. Furthermore, elevated circulating BCAAs levels have been correlated with severity of insulin resistance. It is hypothesized that elevated circulating BCAAs observed in insulin resistance may result from dysregulated BCAAs degradation. Moreover BCAAs metabolism results in an accumulation of toxic BCAAs metabolites, then in turn trigger the mitochondrial dysfunction and stress signaling associated with insulin resistance despite of increment in mitochondrial content and enhancement of oxidative capacity in skeletal muscle [29, 31]. Detailed molecular mechanism of BCAAs overdose on *Fndc5* gene expression in the skeletal and heart muscles remains to be obscure.

Additionally, the synergetic effect of exercise interaction with 20 mg/mL BCAAs supplementation (20BCAA/Ex) on induction of *Fndc5* transcription was evident in our data. We found this interaction had positive effect on *Fndc5* expression and metabolism. Also, we indicated protein content of FNDC5 decreased in 60BCAA as compared with sedentary group. Interestingly, we observed the protein level of FNDC5 in the skeletal muscle was higher in exercised mice in contrast with 20BCAA group. Presumably, this incompatibility between mRNA and protein levels of FNDC5 in the mice is related to the activity of some regulators which affect protein translation [32, 33].

Our findings were in good agreement with Aydin et al. which reported a positive association with metabolism and *Fndc5* gene expression [21]. Unlike our findings, Pekkala et al. indicated no significant up-regulation in *Fndc5* of skeletal muscle in trained individuals [34]. This discrepancy may be mainly due to the type of exercise or simply reflecting differences between human and mice metabolism. The secretory type of *Fndc5* is irisin, which affects total body energy expenditure, as well as browning of WAT (white adipose tissue) [14]. There are several controversy results about *Fndc5* and irisin levels in plasma [13, 14, 16, 21, 35]. Our data showed irisin level was increased in 20BCAA/Ex group. Therefore, we assume that, irisin is increased in response to exercise and 20 mg/mL of dietary BCAAs.

*Pgc-1α*, is a co-activator of various nuclear receptors as well as transcription factors. Previous study has indicated that *Pgc-1α* is involved in regulation of energy metabolism, as well as thermogenesis, in skeletal muscle. In skeletal muscle, *Pgc-1α* regulates not only the mitochondrial function but also metabolic activity of the cell [36, 37]. Tadaishi et al. revealed that, BCAAs metabolism mainly occurs in mitochondria of skeletal muscle and is induced by *Pgc-1α* [36]. However, higher amount of BCAAs (60 mg/mL) reversed such inductive responses, possibly by increased BCAAs catabolic flux which triggers insulin resistance in myoblast [38].

In this study we have examined the effect of exercise and BCAAs supplementation on transcript levels of *Sirt 1 PPARγ, Pgc-1α, Tfam*, *Cox4i1*, *ATP5a1, ATP5b* and *Fndc5* involved in mitochondrial biogenesis and for the first time we have shown that endurance exercise cold increase *ATP5a* and *ATP5b* mRNA expression. In present study, we have demonstrated that *Sirt 1* and *Fndc5* mRNA had a similar trend and they had a positive correlation with each other. *PPARγ* expression was observed in trained mice, emphasizing that exercise could enhance *PPARγ* gene expression [38]. Our results are in agreement with the previous study which indicated *Sirt1* up-regulation and life span extension, and increasing in mitochondrial biogenesis, and ROS production decrease in the skeletal muscle of male mice [10]. One of the limitation of our study was lack of data about no mitochondrial related functional evidences in either tissues, such as mitochondrial number, respiration capacity, β-oxidation to support the physiological observation of BCAAs overload. This is critical issue which should be investigated in further experiments.

According to our knowledge endurance exercise increases the capacity of skeletal muscle for aerobic metabolism through an increase in mitochondrial capacity to generate ATP via oxidative phosphorylation [39], presumably through upregulation of *Pgc-1α* and *PPARγ* expression [40]. Furthermore, BCAAs supplementation may improve muscle fiber size, physical endurance and it may increase mitochondrial activity as already shown that treatment with BCAAs in mice caused significant increase in *Pgc-1α*, *Tfam* and *Sirt1* expression in skeletal muscle. Furthermore, BCAAs supplementation is responsible for antioxidant defense and reduction of ROS [10]. Enhancement of *Pgc-1α* expression triggers the interaction of Pgc-1α with PPARγ, which elaborates the oxidative capacity of mitochondria. On the other hand, Pgc-1α mediates BCAAs metabolism in skeletal muscle through increasing the transcript levels of *branched chain amino acid transaminase 1(Bcat1)*, *Bcat2* and *branched-chain alpha-keto acid dehydrogenase (Bckdh)* [41]. Increment in BCAAs catabolism leads to an increase of cellular Acetyl-CoA, Acetoacetate, and Soccinyl-CoA which reduces glucose oxidation rate and lactate production [36, 39]. Final response of the myoblast to these events is the up-regulation of *Fndc5* through unidentified cascade.

## Conclusion

Our present study suggests that consuming 20 mg/mL BCAAs with exercise (20BCAA/Ex) had positive effect on expression level of gastrocnemius muscle *Fndc5*, *PPARγ*, *Pgc-1α* similar with heart tissue. In addition, Irisin levels were increased at most level after 20 mg/mL BCAAs supplementation which coincided with the exercise (20BCAA/Ex). However, overloading of higher amount of BCAA may have adverse these effects on transcript levels of *Fndc5*. However, physiological aspects of this finding needs to be confirmed in further studies.

## Additional file

Additional file 1: **Figure S1**. Comparative study on amino acid content of proteins in human and mouse. As shown, the assessed proteins were the same as were obtained by STRING analysis (Fig. 2). Dark color indicates more homology in amino acid residues and bright red color represents more dissimilarity between human and mouse proteins. Of interest most similarity was obtained for PPARγ, and FNDC5 between mouse and human. (JPG 86 kb)

## Abbreviations

ANOVA: Analysis of variance
BBB: Blood-brain barrier
BCAAs: Branched chain amino acids
Bcat: Branched chain amino acid transaminase
Bckdh: Branched-chain alpha-keto acid dehydrogenase
Ex: Endurance exercise training
FNDC5: Fibronectin type III domain containing 5
Gapdh: Glyceraldehyde-3-phosphate dehydrogenase
mtDNA: Mitochondrial DNA
mTORC1: Mammalian target of rapamycin complex 1
NRF: Nuclear respiratory factor
Pgc-1*α*: Peroxisome proliferator-activated receptor gamma coactivator 1-alpha
*PPAR*γ: Peroxisome proliferator-activated receptor gamma
qRT-PCR: Quantitative real-time PCR
SEM: Standard error of mean
Sirt1: Sirtuin 1
TFAM: Mitochondrial transcription factor A
TG: Triglyceride
WAT: White adipose tissue
