# Supplementary figures and images for: PPARγ/Pgc-1α-Fndc5 pathway up-regulation in gastrocnemius and heart muscle of exercised, branched chain amino acid diet fed mice
Source: Nutr Metab (Lond). 2018 Aug 29;15:59. doi: 10.1186/s12986-018-0298-3 (PMC6114499; doi:10.1186/s12986-018-0298-3)

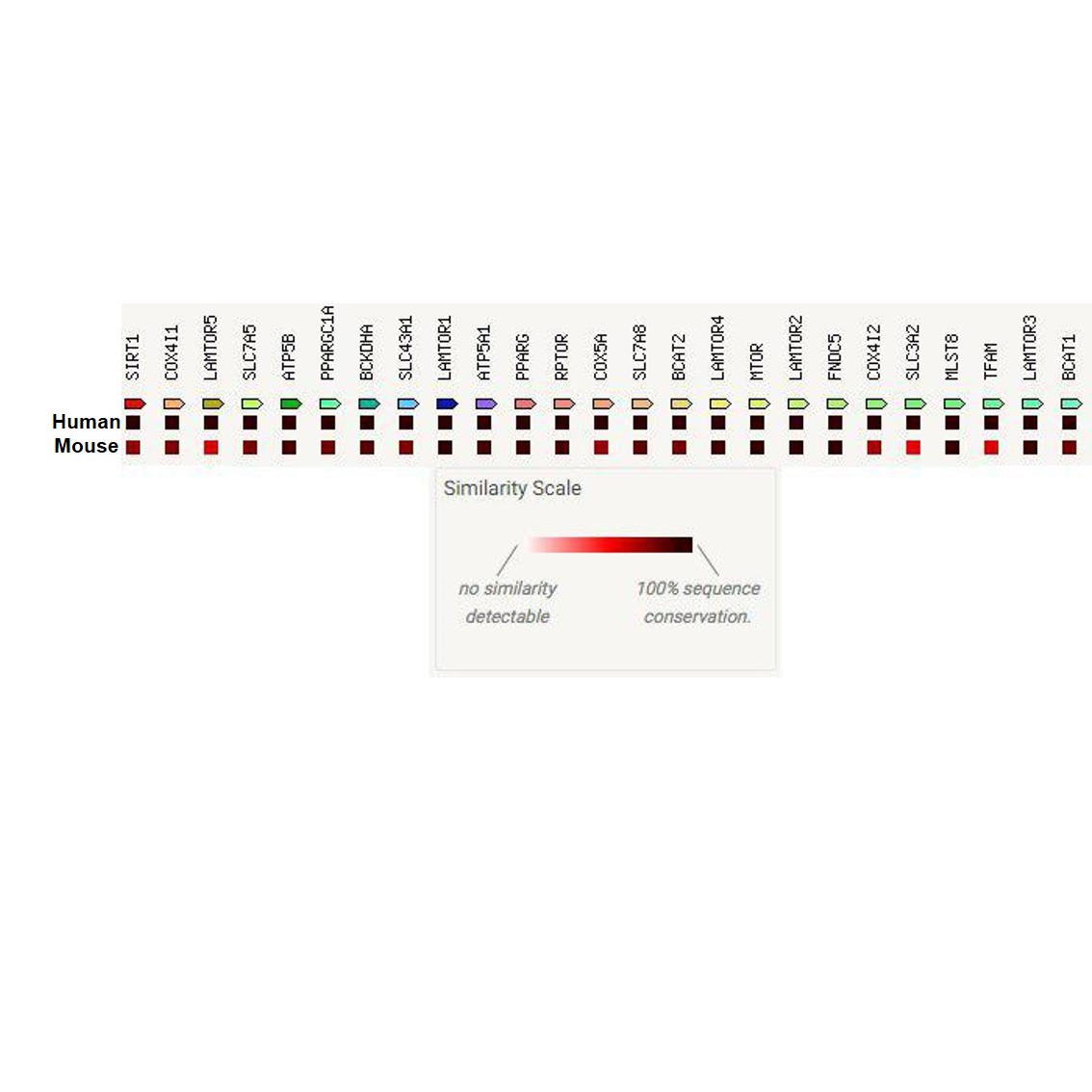

Supplement: Supplementary file 1 — Figure S1. Comparative study on amino acid content of proteins in human and mouse. As shown, the assessed proteins were the same as were obtained by STRING analysis (Fig. 2). Dark color indicates more homology in amino acid residues and bright red color represents more dissimilarity between human and mouse proteins. Of interest most similarity was obtained for PPARγ, and FNDC5 between mouse and human. (JPG 86 kb) [file 12986_2018_298_MOESM1_ESM.jpg]
